# ECG and EEG based detection and multilevel classification of stress using machine learning for specified genders: A preliminary study

**DOI:** 10.1371/journal.pone.0291070

**Published:** 2023-09-01

**Authors:** Apit Hemakom, Danita Atiwiwat, Pasin Israsena

**Affiliations:** 1 Neural Signal Processing Research Team, National Electronics and Computer Technology Center, National Science and Technology Development Agency, Pathumthani, Thailand; 2 Division of Health and Applied Sciences, Faculty of Science, Prince of Songkla University, Hat Yai, Songkhla, Thailand; Vellore Institute of Technology: VIT University, INDIA

## Abstract

Mental health, especially stress, plays a crucial role in the quality of life. During different phases (luteal and follicular phases) of the menstrual cycle, women may exhibit different responses to stress from men. This, therefore, may have an impact on the stress detection and classification accuracy of machine learning models if genders are not taken into account. However, this has never been investigated before. In addition, only a handful of stress detection devices are scientifically validated. To this end, this work proposes stress detection and multilevel stress classification models for unspecified and specified genders through ECG and EEG signals. Models for stress detection are achieved through developing and evaluating multiple individual classifiers. On the other hand, the stacking technique is employed to obtain models for multilevel stress classification. ECG and EEG features extracted from 40 subjects (21 females and 19 males) were used to train and validate the models. In the low&high combined stress conditions, RBF-SVM and kNN yielded the highest average classification accuracy for females (79.81%) and males (73.77%), respectively. Combining ECG and EEG, the average classification accuracy increased to at least 87.58% (male, high stress) and up to 92.70% (female, high stress). For multilevel stress classification from ECG and EEG, the accuracy for females was 62.60% and for males was 71.57%. This study shows that the difference in genders influences the classification performance for both the detection and multilevel classification of stress. The developed models can be used for both personal (through ECG) and clinical (through ECG and EEG) stress monitoring, with and without taking genders into account.

## Introduction

### Stress

Threat or demand causes the body to react physically or mentally, that is, stress. During the period of the reaction, the body’s response, referred to as the “fight-or-flight response,” kicks in, activating the hypothalamus-pituitary-adrenocortical (HPA) axis and sympathetic nervous system (SNS) to enhance physiological responses, such as blood pressure, respiration rate, and heart rate, to prepare the body to face or escape from the threat or demand [1–4]. After dealing with the stressful condition, the parasympathetic nervous system (PNS) is subsequently activated to reduce physiological responses; hence, blood pressure, heart rate, and other physiological parameters are lower as a result. It is beneficial to have momentary stress so that stressful conditions can be effectively dealt with. Prolonged stress, however, often causes various health issues, such as malfunctioning digestive systems, hypertension, major depressive disorder, immunosuppression, etc. [5].

The fight-or-flight response is typically exhibited by both males and females when they encounter stressful situations. For females, however, during different phases (luteal and follicular phases) of the menstrual cycle, they may exhibit different responses to stress [6–8]. During the follicular phase, when the estrogen level peaks, they may tend to exhibit a “tend-and-befriend” response, in which social coping methods are employed to combat stress [9]. This is because estrogen increases parasympathetic control of the heart [10].

Ardell Wellness Stress Test and Stress Coping Resources Inventory [11], Standard Stress Scale [12], and Perceived Stress Scale [13] are questionnaires that are typically used by an expert, in conjunction with an interview, to evaluate one’s stress. Given their subjective nature, such means of stress evaluation can be inaccurate. Effective diagnosis and treatment, therefore, cannot be achieved due to such an inaccurate evaluation. To overcome this problem, neuronal and physiological features from electroencephalogram (EEG) and electrocardiogram (ECG) signals, respectively, have been extracted and used to construct machine learning models in order to detect stress in humans in various studies [15–19, 65–67].

### Heart rate variability

Heart rate variability (HRV)–variation of the cardiac cycle timing–primarily results from the interaction between the PNS and SNS. It is extracted from an ECG trace and investigated in both the frequency and time domains. Examples of HRV features in the time domain are mean heart rate (HR), standard deviation of RR interval (StdRR), the number of times that RR intervals are longer than 20 ms (RR20), and the root mean square of successive RR interval differences (RMSSD). The evaluation of changes in HRV in the frequency domain is typically performed in 2 frequency bands: (i) the high frequency (HF) band, 0.15–0.4 Hz, which results from the activity of the PNS [14]; and (ii) the low frequency (LF) band, 0.04–0.15 Hz, which reflects the interaction between the PNS and SNS.

Machine learning is widely used for stress detection through ECG (HRV) features. In [15], a Random Forest (RF) classifier was trained with Stress index, SD1/SD2, SD2, SD1, LF/HF, HF/(LF+HF), LF/(LF+HF), HF, LF, VLF, mean RR, mean HR, TINN, HRV triangular index, pRR50, RR50, SDSD, RMSSD, StdRR, and features extracted from data recorded with an accelerometer, and provided 78% classification accuracy. In [16], LF, HF, LF/HF, RMSSD, pNN50, pNN20, mean HR, SDNN, and other features extracted from respiration, temperature, and galvanic response (GSR) were used to train logistic regression (LR) and support vector machine (SVM) which respectively resulted in classification accuracy of 80.7% and 82.7%. SVM classifiers and k-Nearest Neighbor kNN (kNN) which were trained using SD2, SD1, LF/HF, normalized HF (HFnorm), normalized LF (LFnorm), HF power, LF power, mean HR, CorrCoef, RMSSD, pNN50, pNN20, NN20, NN50, SDNN, and AVNN produced classification accuracy of 77.78% and 80.56% [17]. HF peak, LF peak, HFnorm, LFnorm, LF/HF, HF, LF, total power, ECG envelope, HRV triangular index, pNN50, NN50, RR, RMSSD, Std HR, mean HR, and SDNN were used to train SVM and kNN classifiers, respectively, providing classification accuracy of 72.82% and 66.52% [18]. A maximum classification accuracy of 94.58% was achieved in [19] using a kNN classifier trained with HF/(LF+HF) skewness, LF/(LF+HF) power, LF/HF mean, HF power, LF power, HR, NN50, RMSSD, and Triangular index.

### Electroencephalogram

The non-invasive recording of electrical potentials on the scalp is referred to as an electroencephalogram (EEG). It is obtained by placing electrodes at several locations on the scalp according to the 10–20 system [20] and feeding electrical signals picked up by the electrodes to a data acquisition unit. The evaluation of EEG is typically performed in five frequency bands: delta 0.01–4.00 Hz, theta 4.01–8.00 Hz, alpha (mu) 8.01–13.00 Hz, beta 13.01–30.00 Hz, and gamma 30.01–100.00 Hz.

EEG and machine learning have been used to detect psychiatric disorders and assess mental performance. Intellectual disability, schizophrenia, and bipolar disorder were detected with accuracies of 97.47%, 94.36%, and 93.49%, respectively, by using kNN trained with EEG features obtained from 69 subjects [21]. Mental performance was assessed using kNN [22], SVM and the greedy algorithm [23]. The classifiers in both the studies were trained with EEG features obtained from 36 subjects. The former achieved 96.77% accuracy, and the latter achieved 97.88% accuracy. The results show the promising potential of using well-known classifiers, such as kNN and SVM, trained with EEG features for mentally related classification problems.

While the brain is at rest, the right and left hemispheres of the brain share equal cognitive workload, where negative emotions are linked with the former and positive emotions with the latter [24, 25]. Deterioration of this balance, however, can be caused by chronic stress. For instance, the right hemisphere of the brains of patients with major depression disorder (MDD) and posttraumatic stress disorder (PTSD) were more active than the left hemisphere, especially for PTSD patients, where such increases in activity were highly observed in the right frontal cortex [26, 27]. Machine learning techniques nowadays use EEG biomarkers to detect stress. In [28], stress induced by a mental arithmetic task (MAT) was 94.79% correctly classified by an SVM classifier trained with prefrontal alpha rhythm. In [29], disparity between the right- and left-hemispheres in the alpha band total power and frontal alpha asymmetry extracted from 32 subjects in the Database for Emotion Analysis using Physiological Signals (DEAP) [30] were employed to train kNN yielding a classification accuracy of 85.63%. In [31], temporal and frontal alpha asymmetry extracted from 33 subjects was used to train SVM which provided 85.20% accuracy for the identification of chronic stress. In [32], low beta waves (13–17 Hz) extracted during eyes closed from 28 participants were used to train Naïve Bayes (NB) which provided 71.43% accuracy for stress classification. In [33], stress while driving was detected using SVM, neural networks (NN), and RF. Four sets of features extracted from 86 participants were used to train these classifiers: 1 set of 6 features in the time domain and 3 sets of features in the frequency domain, which are (i) for each of the 5 frequency bands, power difference between two EEG signals (O1–O2, FC5–FC6, AF3–AF4, F7–F8, P7–P8, F3–F4, and T7–T8); (ii) power ratio within those pairs of EEG signals; and (iii) absolute powers in the 5 frequency bands of each of the 14 electrodes. The resulting classification accuracy was 91.60% (SVM), 89.00% (NN), 87.23% (RF), and 97.95% (ensemble classifier). A more sophisticated feature extraction method called multiscale sample entropy was used in [34] to generate features from EEG signals recorded from 28 subjects with no distinction in genders to train a neural network classifier to quantify levels of enjoyment, thoroughly unrelated to stress, in museum pieces with an accuracy of 98%. We recently proposed ML models for stress detection through 131 ECG and EEG features obtained from 20 subjects whereby some of those features were selected using the filter, wrapper, and hybrid methods for model construction [35]. The highest classification accuracy for low-stress detection was 85% using kNN and RF with only 1 feature (the absolute power of the low gamma band recorded from the T3 electrode), and the highest classification accuracy for high-stress detection was 90% using SVM with 108 features. *Genders*, *however*, *were not considered when building those models*. *Multilevel classification of stress was also not possible using those models*. Although very high classification accuracy of stress detection (96.67%) in different genders was achieved in [36] using SVM through heart rate, systolic blood pressure, diastolic blood pressure, and galvanic skin response, there was a lack of description of the task(s) that the subjects were required to mentally perform in order to induce mental stress for reliable mental stress data collection and ultimately for building the model. In other words, if the subjects were not required to do any mental tasks during data collection, their actual mental conditions at the time of recording were therefore uncontrollable and, most importantly, unknown to the experimenter. Electrodermal activity (EDA) signals obtained from a wearable device were used in [37] to construct an SVM-based stress detection model for both males and females achieving 92.9% detection accuracy. Although the EDA signal reliably reflects the activation of the SNS under arousal obtained from a less invasive device, EDA is a slowly changing signal that has a delay of a few seconds. This limitation makes it unsuitable for stress detection in daily life, which requires real-time responses such as biofeedback. Moreover, the use of different publicly available datasets may be problematic since each company provided the EDA signals obtained from different skin parts, such as wrists, fingertips, or palms. In most cases, EDA signals collected from users in everyday settings may have a low signal-to-noise ratio due to the incorrect placement or displacement of the sensors during the day in the presence of intense activity. These factors are crucial for signal processing approaches. Underlying stress was detected with 66.23% accuracy using a Deep Belief Network (DBN) trained with stress-related physical activity and lifestyle data, such as body mass index (BMI), smoking and drinking behavior, systolic blood pressure, diastolic blood pressure, sleep time, age, and pulse rate [38]. While this work aims to detect the underlying stress from health and nutrition-related datasets obtained from questionnaires which may be subjected to incorrect feedback or superficial responses, our work is designed to evaluate stress based on more reliable and objective physiological indices during a well-known stress induction task. Many studies have shown the correlation between brain activity and heart rate variability during cognitive tasks [39], emotional arousal, and emotional stress [40–44]. *With this association*, *the use of ECG signal-HRV and high temporal-resolution EEG signals is very promising in the development of machine learning models for stress detection*. *Additionally*, *to the best of our knowledge*, *ECG- and EEG-based machine learning models developed specifically for multilevel classification and detection of stress for different genders are still lacking*.

### Objectives

This study aims to develop *machine learning models for detecting the presence of stress*, *i*.*e*., *stress detection*, *from ECG towards real-world use on portable devices*, where currently only 5% of such devices are scientifically validated [45], and *from both ECG and EEG towards clinical use* where a high-quality EEG recording device is available; *and for classifying multiple levels of stress*, *i*.*e*., *multilevel stress classification*, *from both ECG and EEG signals towards also clinical use*. *Our models are capable of detecting and classifying multiple levels of stress for both unspecified (mixed) and specified genders*. *Our models are the first that are capable of detecting and classifying multiple levels of stress using both ECG and EEG for both specified and unspecified genders*. *Specifying genders can help to obtain more reliable classification accuracy and reveal differences in stress levels between females and males*. The models were developed using the Python IDE with scikit-learn machine learning libraries. All the models are achieved by using the filter method to choose ECG and EEG feature subsets extracted from ECG and EEG data acquired from our experiments, where subjects were asked to perform mental arithmetic tasks (MAT). For stress detection, subsets of selected features were employed to train several machine learning algorithms, such as LR, NB, Adaptive Boosting (AdaBoost), SVM, RF, and k-Nearest Neighbor (kNN). The classification accuracy of each classifier was then evaluated. For multilevel stress classification, subsets of selected features were again used to train these individual classifiers. Subsequently, an ensemble technique referred to as “stacking” was employed, whereby classification results from the individual classifiers were used to train the LR classifier, producing the final classification result. The classification accuracy of the stacking technique was evaluated against that of each individual classifier.

## Algorithms

We shall first briefly describe the algorithms used in this study. These algorithms were used because they are widely known and efficient, while their classification mechanisms are distinctively different. This would provide us with a good coverage of approaches to tackling our classification problems.

### Naïve Bayes (NB)

Given a class variable, the value of a particular feature is assumed by the Naïve Bayes (NB) classifier to be independent of the value of any other feature. It is considered attributed by chance if there exists dependency amongst the features [46]. The class with the highest posterior probability is then assigned to a data point [10]. For multiclass classification, the probability for each class is calculated, and then the class with the highest probability is chosen as the final prediction result.

Given an *N*-dimensional feature vector **x**, the probability of class *y*, *P*(*y*|**x**), is calculated by

$$P(y|x)=\frac{P(x|y)P(y)}{P(x)}$$ (1)

The feature vector **x** is then assigned to the class with the maximum a posterior (MAP), which can be computed by

$$y_{MAP}=\underset{y\in Y}{\mathrm{argmax}}\;P(y|x)$$ (2)

$$y_{MAP}=\underset{y\in Y}{\mathrm{argmax}}\frac{P(x|y)P(y)}{P(x)}$$ (3)

$$y_{MAP}=\underset{y\in Y}{\mathrm{argmax}}\;P(x|y)P(y)$$ (4)

The probability *P*(**x**|*y*) is given by

$$P(x|y)=\frac{1}{\sqrt{2\pi \sigma_{y}^{2}}}e^{-\frac{{\left(x-\mu_{y}\right)}^{2}}{2\sigma_{y}^{2}}},$$ (5)

where *μ*_*y*_ and $\sigma_{y}^{2}$ are the mean and variance of all features associated with class *y*, respectively. *P*(*y*) can be obtained by dividing the frequency of each class in the entire dataset by the number of feature vectors. The train and run time complexity of NB are O(*Q*^*N*^∙*Y*) and O(*N*∙*Y*), respectively, where *Q* is the number of feature vectors (data points), *Y* the total classes.

### Logistic Regression (LR)

Logistic Regression (LR) classifier is an extension of linear regression, where a data (feature) vector ***x*** is first fitted into the linear regression model and a logistic function is next applied to restrict the resulting likelihood (probability) value to within the range 0–1 [45, 47–49].

The probability of class 1 given an *N*-dimensional feature vector **x** is calculated by

$$P(y=1|x)=l(\beta^{T}x),$$ (6)

and for class 0 by

$$P(y=0|x)=1-l(\beta^{T}x),$$ (7)

where the likelihood function *l*(**x**) is given by

$$l(x)=\frac{1}{1+e^{-∙x}}$$ (8)

**β** is an (*N*+1)-dimensional coefficient vector, **β** = {*β*_0_,*β*_1_,*β*_2_,…,*β*_*N*_}. For binary classification, if *l*(**β**^*T*^**x**) is greater than 0.5, the feature vector will be determined as class 1 (*y* = 1), otherwise as class 0 (*y* = 0). For multiclass classification, the likelihood value of class *y*, *l*_*y*_(**x**), is given by

$$l_{y}(x)=\frac{e^{\beta_{y}x}}{1+\sum_{y=1}^{Y}e^{\beta_{y}x}},$$ (9)

where ***β***_*y*_ is a zero vector for class 0. The train and run time complexity of LR are O(*Q*∙*N*∙*Y*) and O(*N*), respectively.

### Adaptive Boosting (AdaBoost)

Adaptive Boosting (AdaBoost) classifier is a meta-estimator where multiple classifiers (base estimators) are combined to construct strong classifiers [50]. AdaBoost can be used for both binary and multiclass classification. The algorithm can be formalized in both standard and original forms for both binary and multiclass classification [51, 52]. Decision Tree (DT) classifier is typically used as the base estimator, and the corresponding train and run time complexity are O(*T*∙*Q*∙*N*) and O(*T*), respectively, where *T* is the number of base estimators.

### k-Nearest Neighbor (kNN)

k-Nearest Neighbor (kNN) classifier is a non-parametric approach that finds *k* feature vectors nearest to a given feature vector in *N*-dimensional feature space of the training data and assigns a class to the given feature vector according to the majority of *k* nearest feature vectors [53].

The distance between a given feature vector **x**_1_ and another feature vector **x**_2_ can be computed using the Euclidean distance, given by

$$Distance(x_{1},x_{2})=\sqrt{\sum_{n=1}^{N}{\left(x_{1,n}-x_{2,n}\right)}^{2}},$$ (10)

where *x*_1,*n*_ and *x*_2,*n*_ are coordinates of two feature vectors **x**_1_ and **x**_2_, respectively, in *N*-dimensional feature space. The conditional probability of each class can be computed by

$$P(y=j|X=x)=\frac{1}{k}\sum_{i=1}^{k}I(y_{i}=j),$$ (11)

where *I* is the indicator function (*I* = 1 if *i* = *j*, else *I* = 0). kNN can be used for both binary and multiclass classification. Its train and run time complexity are O(1) and O(*Q*∙*N*), respectively.

### Random Forest (RF)

Random Forest (RF) classification technique is based on the DT technique [33]. In RF, many decision trees are constructed to provide a variety of classification results, and the forest finally predicts a class of a feature vector of interest by taking the majority vote of the classification results given by the trees. See [54] for the formalization of RF. An RF classifier can be built using the total number of features (RF-Max), the Log2 of the number of features (RF-Log2), or the square root of the number of features (RF-Sqrt). RF can be used for both binary and multiclass classification. The train and run time complexity of RF-Max are, respectively, O(*G*∙*Q*∙*N*^2^) and O(*G*∙*N*), RF-Log2 are O(*G*∙*Q*∙(*log*_2_*N*)^2^) and O(*G*∙*log*_2_*N*), RF-Sqrt are O(*G*∙*Q*∙*N*) and $O(G∙\sqrt{N})$, where *G* is the number of decision trees.

### Support Vector Machine (SVM)

Support Vector Machine (SVM) is a non/- linear machine learning algorithm [28, 45, 55]. Features from different classes with the shortest distance between them in an *N*-dimensional feature space are called support vectors. These are used to construct a hyperplane given by

$$w^{T}x+b=0,$$ (12)

and two other hyperplanes which lie on the support vectors and separate two classes are given by

$$w^{T}x+b=1\;and\;w^{T}x+b=-1.$$ (13)

These two hyperplanes are obtained by solving an optimization problem in order to maximize the distance between them, that is, the margin of separation to be $\frac{2}{\left||W|\right|}$. A kernel, the Radial Basis Function (RBF), can be applied in order for SVM to effectively deal with non-linear data. SVM can be used for both binary and multiclass classification. See [56, 57] for the detailed formalization of SVM. The train and run time complexity of SVM are O(*Q*∙*R*) and O(*Q*∙*U*), respectively, where *U* is the number of support vectors, and the train and run time complexity of RBF-SVM are O(*Q*^2^∙*N*+*Q*^3^) and *O*(*U*∙*N*), respectively.

### Stacking

Stacking [58] is an ensemble technique that uses binary or multiclass classification results from several classifiers (*h*_1_(.), *h*_2_(.),…,*h*_*Z*_(.), where *Z* is the number of classifiers) trained with given feature vectors in the first level (base model) as input variables for training a machine learning model on the second level (meta-model, *dh*) to give a final classification result. A stacking model *s*_*Z*_ can then be defined as

$$s_{Z}(.)=dh(h_{1}(.)\times h_{2}(.),h_{1}(.)\times h_{3}(.),\ldots ,h_{Z-1}(.)\times h_{Z}(.)),$$ (14)

where *h*_*b*_(.)×*h*_*c*_(.) is the pairwise multiplication of the results from the base models. The train and run time of a stacking model are $\sum_{z=1}^{Z}O_{train,h_{z}}(.)+O_{dh}$ and $\sum_{z=1}^{Z}O_{run,h_{z}}(.)+O_{dh}$, respectively, where $O_{train,h_{z}}(.)$ and $O_{run,h_{z}}(.)$ are the train and run time of each base model.

### Filter method for feature selection

To achieve successful classification, it is not crucial to use a substantial number of features, because some features might be redundant and weakly relevant, or noisy and completely irrelevant. Only features that are strongly or weakly relevant and non-redundant should be used for training [59]. Generalization, scalability, understandability, and prediction performance can be enhanced through feature selection, which also provides a faster and more cost-effective model and reduces computational complexity and storage [60]. The so-called filter and wrapper methods are widely used for feature selection. Intrinsic characteristics of features prior to learning tasks are examined by the filter method. This method, as a result, is computationally fast, efficient, and independent of the learning algorithm. On the other hand, the wrapper method uses the classification performance of a machine learning algorithm to evaluate features. As a result, the learning algorithm of choice influences the features selected. This technique is also more computationally expensive and more prone to overfitting compared to the filter method. This study employed the filter method for feature selection due to its efficiency and fast computation.

### Performance evaluation

The overall classification performance of a classifier can be quantified through accuracy, area under the ROC curve (AUC), and F1 score. The accuracy of a classifier shows the ratio between correctly classified cases (for both positive and negative classes) and all cases. The AUC essentially shows the ability of a model to correctly distinguish classes; that is, the higher the AUC, the better the model is at correctly predicting classes. The AUC values range from 0 to 1, with 1 indicating perfect classification, 0 all classes incorrectly classified, and 0.5 all classes indistinguishable. The F1 score is defined as the harmonic mean of precision and recall, focusing only on the positive class. The F1 score values range from 0 to 1, with 1 indicating perfect classification and 0 indicating all classes incorrectly classified. The accuracy and F1 score are given by

$$Accuracy=\frac{true\;positive+true\;negative}{true\;positive+true\;negative+false\;positive+false\;negative}*100,$$ (15)

$$F1\;score=2*\frac{recall*precision}{recall+precision},$$ (16)

$where\;recall=\frac{true\;positive}{true\;posotive+false\;negative},\;and\;precision=\frac{true\;positive}{true\;positive+false\;positive}$.

## Materials and methods

### Data collection

The experimental protocol was accredited by the Human Research Ethics Committee of Prince of Songkla University, Songkhla, Thailand (protocol number: 62-029-1-1, date of approval: 22^nd^ July 2020). Subjects were recruited between August 2020 and May 2021. Forty healthy university students, including 21 females (18–25 years of age), were selected for this study. Written consents from the subjects were obtained. Only one of the authors who performed data collection has access to information that can identify individual participants during and after data collection. Each participant is identified using a specific code, and the data are stored on an encrypted computer to which the only one author has access. All subjects have no history of neurological or cardiovascular diseases and no history of using any medication that might affect the autonomic nervous system. The female subjects were asked to provide the date of the first day of their last period to determine the phase of the menstrual cycle that they were in on the recording day. After a rest period of 15 minutes, their blood pressure was measured. The Thai version of the Perceived Stress Scale (T-PSS-10) [61] was used to measure underlying stress in the last month before the experimental day, from which a subject’s stress was classified into three levels. All 40 subjects taking part in this experiment had low or moderate stress scores and thus were included in the study. All experiments were conducted in quiet laboratory conditions in the afternoon (2–4 p.m.) to control the effect of circadian rhythms on physiological activity.

The ECG was acquired using a bipolar limb lead (right arm-left leg) connected to an ECG amplifier (ADInstruments, Dunedin, New Zealand). The signals were band-pass filtered between 0.3 and 200 Hz and sampled at 1000 Hz. EEG electrodes were placed using a cap with 8 electrodes: Fp1, Fp2, F3, F4, P3, P4, T3, and T4 according to the international 10–20 system. Monopolar recording was performed between the active electrode and the reference electrode (average mastoids). The sampling frequency was 2000 Hz, and low-pass filtering with a 200 Hz cut-off frequency was applied. The impedance was kept at ≤ 10 kΩ (eego™ 8 ANT neuro, Hengelo, Netherlands).

**Fig 1:**
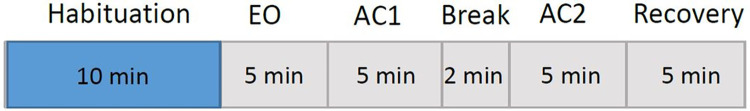
Experimental procedure. The experiment comprised 6 periods: 1) Habituation, 2) non-stress with eyes open (EO), 3) performing arithmetic tasks under low-stress condition (AC1), 4) break, 5) performing arithmetic tasks under high-stress condition (AC2), and 6) Recovery.

Mental arithmetic task (MAT), which is widely used to induce brain activation and physiological stress responses (cortisol levels, blood pressure, and escalated heart rate) [62–68], was used to induce stress in this study. A within-subjects design in which all participants are exposed to both control and stress conditions is used in this experiment to help reduce errors associated with interindividual differences in physiological factors such as breathing rate, heart rate, and blood pressure. The experiment protocol was performed in six steps (see Fig 1). Step 1 (Habituation): A brief introduction was given to the subjects to familiarize them with the experimental procedure. No EEG or ECG signal was recorded during this period. Step 2 (Eyes open: EO period, that is, non-stress condition or baseline): baseline EEG and ECG signals were measured for 5 minutes. Subjects were instructed to look at a fixation dot on the computer screen with minimal head and body movement. Step 3 (Arithmetic stress task 1: AC1 period, that is, low stress condition): The subjects performed mental arithmetic tasks presented on the computer screen, which involved subtraction (-), addition (+), division (/), and multiplication (x) between four numbers in a quiet environment for 5 minutes. The subjects completed arithmetic problems presented one at a time. After a problem appeared on the screen, the subjects had 30 seconds to give the correct answer by clicking the mouse as quickly as possible. Feedback (correct or incorrect) was provided immediately following each response.

The difficulty and time given to each arithmetic problem were set in such a way that the subjects could not exceed an accuracy of 50% (as derived from pilot subjects). Step 4 (Break): subjects were allowed to relax for 2 minutes. Step 5 (Arithmetic stress task 2: AC2 period, that is, high stress condition): the subjects performed the same procedure as described in the third step but with the audio distraction of a human voice talking. The audio distraction was used to reduce cognition while in mental demand to augment distress [54] and to mimic the real work environment, which is typically interrupted by sensory demand (stress detection in working people). Step 6 (Recovery): the subjects were allowed to recover for 5 minutes.

### Feature extraction

The ECG signals were further digital high-pass filtered at 1 Hz and examined to exclude cardiac arrhythmias–bradycardia (heart rate, HR, < 60), tachycardia (HR > 100), premature contraction. Mean HR (beats per minute) and the following heart rate variability features were then extracted: RMSSD (ms), pNN50 (%), NN50 (beats), SDNN (ms), AVNN (ms), LF/HF Ratio, HF Norm (a.u.), LF Norm (a.u.), LF (0.04–0.15 Hz) (ms2), HF (0.15–0.4 Hz) (ms2) (LabChart Software, ADInstruments). EEG signals were further digital high-pass filtered at 1 Hz. The 50 Hz power and corresponding harmonics were notch filtered using an FIR filter with a +/- 1 Hz cutoff. The fast Fourier transform (FFT) was used to calculate the EEG absolute power of 7 frequency bands: delta 0–4 Hz, theta 4–8 Hz, alpha (mu) 8–13 Hz, beta1 13–20 Hz, beta2 20–30 Hz, low gamma 30–60 Hz, and high gamma 60–100 Hz from the 8 electrode positions. Relative power is the ratio between the absolute power of a frequency band and the total power. Additionally, the interhemispheric asymmetries of the absolute and relative power of alpha, beta1, and beta2 were also calculated by taking the difference between the amplitudes of the signals and then normalizing it to the sum of their amplitudes in 4 homologous electrode pairs: T3-T4 (temporal cortex), P3-P4 (parietal cortex), F3-F4 (frontal cortex), and Fp1-Fp2 (prefrontal cortex). In summary, EEG features used in this study consisted of 1) the absolute and relative power of 7 frequency bands * 8 electrode positions, making up a total of 112 features; and 2) interhemispheric asymmetry of the absolute and relative power of 3 bands * 4 electrode pairs, making up a total of 24 features. The total number of ECG features was 11, and EEG was 136 (147 in total). All the extracted features from all the conditions, including the baseline, can be downloaded from [69] (doi: 10.5061/dryad.kd51c5bbf).

### Selection of features

The number of feature(s) in subsets ranged from 1 to 10 when using only ECG features to train and validate our models and from 1 to 146 when using both ECG and EEG features. The filter method was used to perform feature selection to reduce the number of features, whereby an ANOVA F-value was employed to iteratively examine ranks of feature subsets of a certain size (1–10 for ECG, 1–146 for ECG and EEG) through the degree of linear dependency between two groups of random variables, that is, a given feature subset and classes. ANOVA was suitable for this task, since we had multiple categorical independent variables, i.e., classes. It is therefore more efficient for the concurrent estimation of dependencies between a feature subset and multiple classes. On the other hand, using a pair-wise t-test would require extensive operation of the Bonferroni test to lower the likelihood of obtaining false-positive findings (Type I errors). After feature subsets of a certain size had been ranked, the highest-ranking feature subset of a certain size was fed into a classification model for training and testing, and classification performances were determined. Note that the goal was to achieve the highest classification accuracy using a validated certain size of feature subsets.

### Stress detection and multilevel stress classification

#### Stress detection

**Fig 2:**
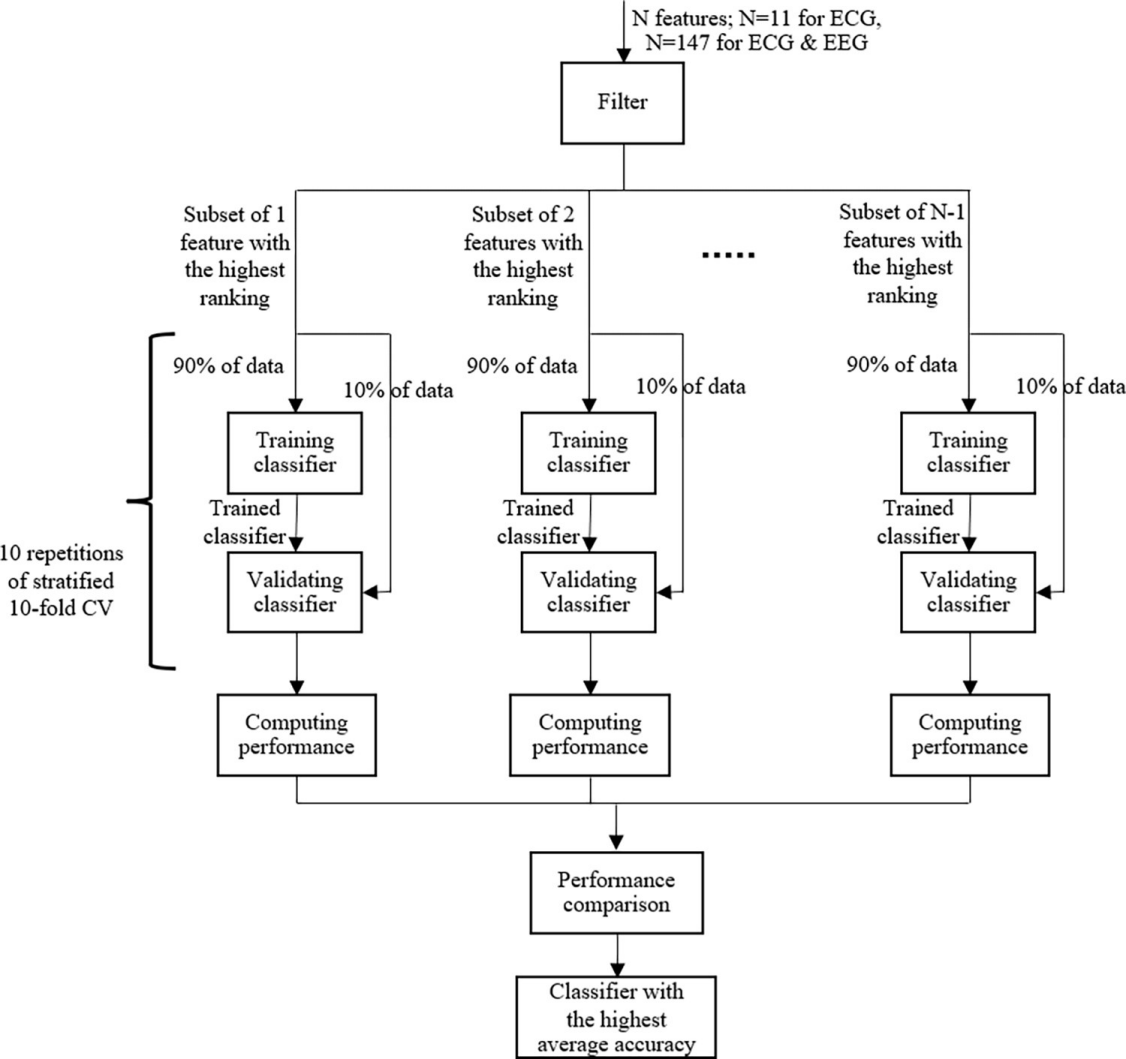
Workflow diagram for building a classifier. Each classifier was trained and validated using different subsets of selected features, whereby 10-fold cross validation was performed to prevent overfitting.

Subsets of selected ECG features were used to train and validate NB, LR, AdaBoost, kNN, RF, SVM, and RBF-SVM binary classifiers in a cross-validation manner to detect the presence of stress in 3 separate scenarios: (i) the presence of low stress–classification between no stress (EO period, Class 0) and low stress (AC1 period, Class 1) conditions; (ii) the presence of high stress–classification between no stress (EO period, Class 0) and high stress (AC2 period, Class 2) conditions; and (iii) the presence of stress without any indication of its level–classification between no stress (EO period, Class 0) and (low & high combined) stress conditions (features for both AC1 and AC2 periods were labeled as Class 1). To prevent overfitting, stratified 10-fold cross validation was performed by splitting (a selected subset of features of) 40 subjects into 10 subsets, whereby 9 subsets of them, containing a selected subset of features, were used to train our classifiers and the remaining set, containing a selected subset of features, to validate the classifiers to obtain classification accuracy, area under the ROC curve (AUC), and F1 score, and the splitting was repeated 10 times to obtain an average classification accuracy. Next, 10 trials of cross validation were performed to obtain a final average classification accuracy, AUC, and F1 score by averaging all the accuracy, AUC, and F1 score values, respectively, from all the 10 trials of the cross validation. A workflow diagram for building a classifier is shown in Fig 2. GridSearchCV in scikit-learn was used to perform stratified 10-fold cross validation and to identify the best parameters of kNN, SVM, and RBF-SVM. For Scenarios 1 and 2, the best values of k for kNN ranging from 1 to 71 for the mixed (unspecified) genders, from 1 to 37 for the females, and from 1 to 33 for the males with a step size of 2 were searched using GridSearchCV to find the values providing the highest classification accuracy. For Scenario 3, the best values of k for kNN ranging from 1 to 107 for mixed genders, from 1 to 55 for the females, and from 1 to 51 for the males with a step size of 2 were searched using GridSearchCV. Note that only odd values of k in the specified ranges were considered. For SVM and RBF-SVM, the best values of C between 0.0001 and 10000 with a step size of 10x were searched using GridSearchCV in all the scenarios, and the best values of gamma for RBF-SVM ranging from 0.0001 to 1 with a step size of 10x were searched using GridSearchCV. The DT classifier was used as the base estimator for AdaBoost, and the number of base estimators was 100. RF-Max, RF-Log2, and RF-Sqrt used 100 DTs. *It should be noted that this work is different from our previous work [35], as this work uses more data from a larger number of subjects, more features, more classification algorithms, and takes genders into account for building stress detection models, resulting in entirely different stress detection models.*

These classifiers were first trained and validated using only ECG features (detailed results are shown in Tables 1–3). They were then trained and validated using both ECG and EEG features to enhance classification performance (see results in Table 4).

**Table 1:** Classification results of classifiers for differentiating between the non-stress (EO) and low-stress (AC1) periods from ECG, the number of features and parameters used to obtain highest accuracy, where for brevity Ave. = average, Acc. = accuracy, and Std. = standard deviation.

	Algorithm	Ave. Acc. (%)	Std. of Acc. (%)	Highest Acc. (%)	Number of features	Parameter	Ave. AUC	Ave. F1-score
**Mixed genders**	**ADAB**	62.38	2.05	63.75	2		0.61	0.62
	***kNN***	*69*.*13*	*1*.*86*	*72*.*50*	*2*	*k = 5*	0.70	0.69
	**LR**	65.00	1.12	66.25	3		0.66	0.65
	**NB**	62.75	0.94	63.75	2		0.63	0.61
	**RF-Log2**	61.38	2.05	65.00	3		0.63	0.59
	**RF-Max**	60.38	2.02	63.75	4		0.63	0.59
	**RF-Sqrt**	59.38	1.15	61.25	1		0.64	0.59
	**SVM**	65.88	0.80	67.5	5	C = 0.0001	0.70	0.65
	**RBF-SVM**	66.38	0.88	67.50	3	C = 0.0001, gamma = 0.0001	0.69	0.65
**Females**	**ADAB**	61.55	3.06	68.00	1		0.64	0.57
	***kNN***	*70*.*45*	*2*.*22*	*73*.*50*	*6*	*k = 5*	0.76	0.67
	**LR**	64.55	1.19	67.00	6		0.71	0.62
	**NB**	62.85	1.78	66.00	1		0.70	0.60
	**RF-Log2**	63.50	1.92	66.50	2		0.75	0.60
	**RF-Max**	62.10	2.62	67.50	3		0.73	0.58
	**RF-Sqrt**	63.90	2.75	69.50	3		0.74	0.59
	**SVM**	69.25	1.21	71.50	11	C = 10	0.76	0.67
	**RBF-SVM**	69.30	1.55	71.50	5	C = 1000, gamma = 0.01	0.78	0.68
**Males**	**ADAB**	58.83	3.14	65	3		0.67	0.55
	***kNN***	*68*.*25*	*2*.*67*	*74*.*17*	*2*	*k = 3*	0.69	0.66
	**LR**	61.42	1.49	64.17	1		0.69	0.59
	**NB**	61.50	2.78	65.83	5		0.67	0.57
	**RF-Log2**	59.17	2.37	64.17	2		0.62	0.52
	**RF-Max**	56.67	2.76	62.50	1		0.60	0.52
	**RF-Sqrt**	58.50	2.32	61.67	5		0.60	0.53
	**SVM**	64.42	2.53	66.67	3	C = 0.0001	0.68	0.60
	**RBF-SVM**	63.92	1.54	66.67	4	C = 10, gamma = 0.1	0.69	0.62

**Table 2:** Classification results of classifiers for differentiating between the non-stress (EO) and high-stress (AC2) periods from ECG, the number of features and parameters used to obtain highest accuracy, where for brevity Ave. = average, Acc. = accuracy, and Std. = standard deviation.

	Algorithm	Ave. Acc. (%)	Std. of Acc. (%)	Highest Acc. (%)	Number of features	Parameter	Ave. AUC	Ave. F1-score
**Mixed genders**	**ADAB**	67.75	2.00	68.75	7		0.78	0.67
	***kNN***	*73*.*25*	*0*.*83*	*73*.*75*	*5*	*k = 65*	0.77	0.73
	**LR**	70.63	0.84	71.25	5		0.75	0.70
	**NB**	70.38	1.38	72.50	5		0.74	0.69
	**RF-Log2**	61.75	1.39	65.00	11		0.65	0.60
	**RF-Max**	64.38	1.61	66.25	7		0.70	0.61
	**RF-Sqrt**	61.50	1.35	63.75	10		0.66	0.59
	**SVM**	72.88	0.80	73.75	5	C = 0.0001	0.76	0.72
	**RBF-SVM**	73.00	0.83	73.75	5	C = 0.0001, gamma = 0.0001	0.76	0.73
**Females**	**ADAB**	66.40	2.72	71.00	1		0.75	0.65
	***kNN***	*77*.*20*	*1*.*36*	*79*.*00*	*2*	*k = 3*	0.82	0.75
	**LR**	69.45	0.65	70.5	2		0.78	0.67
	**NB**	69.90	1.02	71.50	1		0.78	0.68
	**RF-Log2**	67.10	2.85	73.00	1		0.73	0.64
	**RF-Max**	67.45	2.65	72.50	1		0.75	0.63
	**RF-Sqrt**	65.60	3.51	71.50	11		0.73	0.62
	**SVM**	74.35	1.45	78.50	8	C = 10000	0.82	0.72
	**RBF-SVM**	76.50	2.05	81	11	C = 10000, gamma = 0.01	0.84	0.73
**Males**	**ADAB**	63.83	3.36	70.00	7		0.69	0.59
	***kNN***	*71*.*17*	*1*.*59*	*74*.*17*	*5*	*k = 31*	0.77	0.68
	**LR**	66.58	1.08	68.33	4		0.77	0.63
	**NB**	65.42	1.36	67.50	3		0.74	0.62
	**RF-Log2**	53.17	2.66	58.33	8		0.56	0.49
	**RF-Max**	56.25	3.30	61.67	9		0.58	0.54
	**RF-Sqrt**	54.50	1.87	56.67	7		0.60	0.51
	**SVM**	65.50	1.55	69.17	4	C = 10	0.76	0.62
	**RBF-SVM**	67.83	0.85	69.17	7	C = 1, gamma = 0.1	0.77	0.66

**Table 3:** Classification results of classifiers for differentiating between the non-stress (EO) and (low & high combined) stress (AC1 combined with AC2) periods from ECG, the number of features and parameters used to obtain highest accuracy, where for brevity Ave. = average, Acc. = accuracy, and Std. = standard deviation.

	Algorithm	Ave. Acc. (%)	Std. of Acc. (%)	Highest Acc. (%)	Number of features	Parameter	Ave. AUC	Ave. F1-score
**Mixed genders**	**ADAB**	69.58	1.13	71.67	11		0.69	0.64
	**kNN**	73.25	0.87	74.17	2	k = 19	0.73	0.66
	**LR**	68.42	0.69	70.00	10		0.71	0.50
	**NB**	71.42	0.75	71.67	4		0.70	0.65
	**RF-Log2**	69.08	1.20	70.83	7		0.68	0.61
	**RF-Max**	69.50	1.00	71.67	7		0.69	0.61
	**RF-Sqrt**	68.75	1.13	70.83	7		0.68	0.62
	***SVM***	*74*.*75*	*0*.*65*	*75*.*83*	*10*	*C = 1000*	0.73	0.65
	**RBF-SVM**	74.25	1.08	75.83	11	C = 10000, gamma = 0.01	0.72	0.66
**Females**	**ADAB**	74.45	1.56	76.67	3		0.77	0.68
	**kNN**	78.48	0.85	79.76	3	k = 11	0.76	0.68
	**LR**	77.21	1.05	79.05	5		0.75	0.67
	**NB**	72.10	0.93	73.81	5		0.73	0.66
	**RF-Log2**	76.55	1.67	79.76	3		0.78	0.70
	**RF-Max**	76.71	0.97	78.33	2		0.77	0.68
	**RF-Sqrt**	77.45	2.29	80.71	3		0.80	0.69
	**SVM**	78.26	0.41	79.29	10	C = 10	0.80	0.70
	***RBF-SVM***	*79*.*81*	*0*.*78*	*81*.*19*	*9*	C = 1000, gamma = 0.01	0.84	0.73
**Males**	**ADAB**	67.13	1.58	70.33	2		0.66	0.60
	***kNN***	*73*.*77*	*0*.*82*	*75*.*00*	*4*	k = 1	0.72	0.66
	**LR**	66.87	0.50	68.33	5		0.69	0.44
	**NB**	66.70	0.85	68.33	2		0.66	0.56
	**RF-Log2**	65.97	1.52	68.00	8		0.66	0.56
	**RF-Max**	67.67	1.18	70.00	7		0.65	0.55
	**RF-Sqrt**	66.37	2.51	70.33	8		0.65	0.55
	**SVM**	68.13	1.97	72.00	10	C = 10000	0.72	0.55
	**RBF-SVM**	68.60	1.74	71.67	4	C = 10000, gamma = 1	0.70	0.56

**Table 4:** Best classification results for differentiating between the non-stress (EO) and low-stress (AC1) periods, non-stress (EO) and high-stress (AC2) periods, and (EO) and (low & high combined) stress (AC1 combined with AC2) periods from ECG and EEG, the number of features and parameters used to obtain highest accuracy, where for brevity Std. = standard deviation.

		Mixed genders	Females	Males
**No stress vs Low stress**	**Average accuracy (%)**	86.13	91.40	*90*.*08*
	**Std. of accuracy (%)**	0.67	2.19	*0*.*69*
	**Highest accuracy (%)**	87.5	95.00	*91*.*67*
	**Number of features**	24	18	*4*
	**Algorithm**	SVM	SVM	*RBF-SVM*
	**Parameter**	C = 100	C = 100	*C = 10*, *gamma = 1*
	**Average AUC**	0.90	0.98	0.96
	**Average F1-score**	0.86	0.92	0.88
**No stress vs High stress**	**Average accuracy (%)**	*87*.*75*	*92*.*70*	87.58
	**Std. of accuracy (%)**	*0*.*75*	*1*.*03*	1.42
	**Highest accuracy (%)**	*88*.*75*	*95*.*00*	90.00
	**Number of features**	*9*	*11*	7
	**Algorithm**	*SVM*	*SVM*	kNN
	**Parameter**	*C = 1*	*C = 10*	k = 9
	**Average AUC**	0.96	0.99	0.98
	**Average F1-score**	0.88	0.91	0.86
**No stress vs (low&** **high) Stress**	**Average accuracy (%)**	87.50	90.95	88.87
	**Std. of accuracy (%)**	0.65	0.68	1.14
	**Highest accuracy (%)**	88.33	92.14	89.67
	**Number of features**	42	68	5
	**Algorithm**	SVM	RBF-SVM	kNN
	**Parameter**	C = 10	C = 100, gamma = 0.1	k = 9
	**Average AUC**	0.93	0.98	0.96
	**Average F1-score**	0.86	0.89	0.85

#### Multilevel stress classification

*While our previous work [35] proposed only models for stress detection without taking genders into account, this work proposes new models for multilevel stress classification with genders being considered.* Subsets of selected features were used to individually train and validate NB, LR, AdaBoost, kNN, RF, SVM, and RBF-SVM multilevel classifiers that simultaneously distinguish 2 levels of stress (low stress–Class 1, or high stress–Class 2) from the baseline (no stress, Class 0). The best values of k for kNN ranging from 1 to 107 for the mixed genders, from 1 to 55 for the females, and from 1 to 51 for the males, with a step size of 2, were searched using GridSearchCV. Note that only odd values of k were considered. GridSearchCV with a step size of 10x was used to search for the best values of C for SVM and RBF-SVM ranging from 0.0001 to 10,000 and the best values of gamma for RBF-SVM ranging from 0.0001 to 1. The DT classifier was used as the base estimator for AdaBoost, and the number of base estimators was 100. RF-Max, RF-Log2, and RF-Sqrt used 100 DTs. Ten trials of stratified 10-fold cross validation were performed in the same manner as in the training and validation of the stress detection models, and a final average classification accuracy, AUC, and F1 score were determined.

For the stacking technique, classification results from all the individual classifiers with different settings were served as input for training an LR classifier, from which the final classification results were obtained, whereby stratified 10-fold cross validation was carried out and repeated 10 times to obtain a final average classification accuracy, AUC, and F1 score. The best values of k for kNN ranging from 11 to 39, with a step size of 2, were searched using GridSearchCV. GridSearchCV with a step size of 10x was used to search for the best values of C for SVM and RBF-SVM ranging from 0.0001 to 10,000 and the best values of gamma for RBF-SVM ranging from 0.0001 to 1.

## Results

### Stress detection

Tables 1–3 show the detailed classification accuracy of each classifier and scenario using ECG features only, together with the AUC and F1 score. For Scenario 1 (Table 1), kNN provided the highest average classification accuracy for low stress detection in the mixed genders (69.13%), females (70.45%), and males (68.25%), respectively. Note that the average classification accuracy for the females was only 2% higher than that for the males. For Scenario 2 (Table 2), kNN again provided the highest average classification accuracy for high stress detection in the mixed genders (73.25%), females (77.20%), and males (71.17%), respectively. For Scenario 3 (Table 3), SVM, RBF-SVM, and kNN provided the highest classification accuracy for (low & high combined) stress detection in the mixed genders (74.75%), females (79.81%), and males (73.77%), respectively. For Scenarios 2 and 3, there were marked differences of approximately 6% in the average classification accuracy between the females and the males. Regarding the average AUC and F1 score, the classifiers with the highest average classification accuracy also yielded the highest or second highest average AUC and F1 score among all the classifiers for the same types of genders in all the scenarios.

Table 4 shows the classification accuracy of stress detection using both ECG and EEG features, together with the AUC and F1 score. For the mixed genders, the average classification accuracy substantially increased to 86.13% for low-stress detection using SVM, to 87.75% using SVM for high-stress detection, and to 87.50% using SVM for (low & high combined) stress detection. For the females, the average classification accuracy also markedly increased to 91.40% using SVM for low-stress detection, to 92.70% using SVM for high-stress detection, and to 90.95% using RBF-SVM for (low & high combined) stress detection. For the males, the average classification accuracy significantly increased to 90.08% using RBF-SVM for low-stress detection, to 87.58% using kNN for high-stress detection, and to 88.87% using kNN for (low & high combined) stress detection. In all the cases, the average AUC and F1 score also significantly increased. These increases reflect that EEG activities were highly associated with stress, hence helping improve the classification accuracy. Note that in all the scenarios, the average classification accuracy for the females was higher (1.32%, 5.12%, and 2.08% for Scenarios 1, 2, and 3, respectively) than that for the males.

### Multilevel stress classification

**Fig 3:**
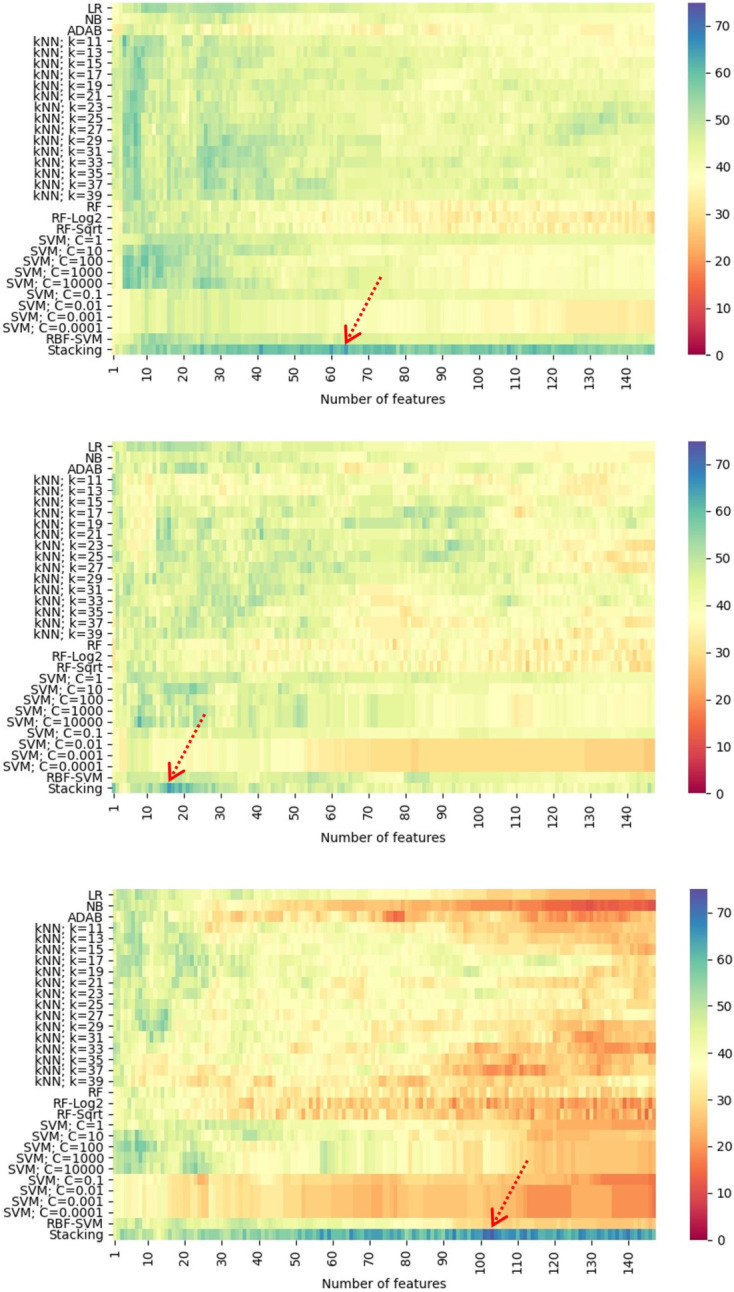
Highest classification accuracy (%) for multilevel stress classification from ECG and EEG of (a) the mixed genders, (b) females, and (c) males using individual classifiers with different parameters and the stacking technique. The arrows point to the numbers of features used in the stacking technique providing the highest classification accuracy.

When performing multilevel stress classification from ECG and EEG using individual classifiers, the average classification accuracy was only 61.00% using SVM, 61.62% using kNN, and 61.57% using SVM for the mixed genders, females, and males, respectively, as shown in Table 5. Using the stacking techniques (see Table 6) improved the average classification accuracy for the mixed genders, females, and males to 64.08%, 62.60%, and 71.57%, respectively. The average AUC for all the genders also increased. It must be noted that in every trial, the stacking technique outperformed every individual classifier. Fig 3(A)–3(C) show the highest classification accuracy obtained using the stacking technique.

**Table 5:** Best classification results for multilevel stress classification from ECG and EEG using individual classifiers, the number of features and parameters used to obtain highest accuracy, where for brevity Std. = standard deviation.

		Mixed genders	Females	Males
**No stress vs. low stress vs. high stress**	**Average accuracy (%)**	61.00	61.62	61.57
	**Std. of accuracy (%)**	0.82	0.90	2.45
	**Highest accuracy (%)**	62.50	62.38	67.00
	**Number of features**	9	27	10
	**Algorithm**	SVM	kNN	SVM
	**Parameter**	C = 100	k = 31	C = 100
	**Average AUC**	0.78	0.80	0.79
	**Average F1-score**	0.59	0.61	0.59

**Table 6:** Classification results for multilevel stress classification from ECG and EEG using the stacking technique in relation to Fig 3(A)–3(C).

		Mixed genders	Females	Males
**No stress vs. low stress vs. high stress**	**Average accuracy (%)**	64.08	62.60	*71*.*57*
	**Std. of accuracy (%)**	0.45	1.55	*1*.*04*
	**Highest accuracy (%)**	65.00	65.24	*73*.*33*
	**Number of features**	64	16	*103*
	**Average AUC**	0.81	0.81	0.83
	**Average F1-score**	0.63	0.58	0.68

Tables 7 and 8 show performance comparisons between this study and others. It should be noted that since there does not exist standardization of datasets, it is not straightforward to obtain a fair comparison among studies. Also, the types and use of stressors, and the data acquisition device significantly impact training data.

**Table 7:** Performance comparison of different stress detection methods based on ECG.

Study	Number of subjects	Length		Gender	Accuracy (%)
**[49]**	16	30 minutes		Mixed	90.62
**[70]**	46	10 minutes		Mixed	74.60
**[71]**	12	3 minutes		Mixed	79.00
**Proposed**	40	5 minutes	low stress	Mixed	69.13
				Female	70.45
				Male	68.25
			high stress	Mixed	73.25
				Female	77.20
				Male	71.17
			low&high	Mixed	74.75
				Female	79.81
				Male	73.77

**Table 8:** Performance comparison of different stress detection and multilevel stress classification (MC) methods based on EEG and/or other physiological signals, where brevity ls. = low stress, hs. = high stress, lhs. = low&high stress, pb. = data taken from publicly available dataset.

Study	Number of subjects	Signal	Task	Classifier	Gender	Accuracy (%)
**[54]**	7	EEG	Detection	LDA	Mixed	82.60
**[72]**	12	EEG	Detection	SVM	Mixed	79.00
**[73]**	32 ^pb^	EEG	Detection	kNN	Mixed	73.38
**[74]**	22	EEG & fNIRS	Detection	SVM	Male	91.70
**[75]**	11	EEG	MC	SVM	Male	80.32
**[76]**	20	EEG	MC	SVM	Mixed	65.00
**[77]**	26	EEG	MC	kNN	Mixed	90.90
**[78]**	28	EEG	MC	SVM	Mixed	78.57
**[79]**	4	EEG & fNIRS	MC	NB	Mixed	36.50
**[80]**	35	EEG	MC	SVM	Mixed	65.00
**[81]**	15 ^pb^	BVP, EDA, Tsk, & ACC	Detection, MC	DCNN	Mixed	99.80, 99.50
**[82]**	15 ^pb^	ACC, ECG, BVP, Tsk, Resp, EMG, & EDA	Detection, MC	DANN	Mixed	95.21, 84.32
**[83]**	20	ECG, voice, & facial expression	Detection	DCNN	Mixed	85.10
**Proposed**	40	EEG, ECG	Detection	SVM	Mixed	86.13 (ls.)
				SVM	Female	91.40 (ls.)
				RBF-SVM	Male	90.08 (ls.)
				SVM	Mixed	87.75 (hs.)
				SVM	Female	92.70 (hs.)
				kNN	Male	87.58 (hs.)
				SVM	Mixed	87.50 (lhs.)
				RBF-SVM	Female	90.95 (lhs.)
				kNN	Male	88.87 (lhs.)
			MC	SVM	Mixed	61.00
				kNN	Female	61.62
				SVM	Male	61.57
				Stacking	Mixed	64.08
					Female	62.60
					Male	71.57

*fNIRS: Functional near-infrared spectroscopy, BVP: Blood volume pressure, EDA: Electrodermal activity, Tsk: Skin temperature, ACC: Accelerometer, Resp: Respiration, EMG: Electromyogram, DCNN: Deep learning convolutional neural network, DANN: Deep learning artificial neural network

The classification accuracy of our models for stress detection from ECG is comparable to other studies [49, 70, 71] (see Table 7) in all the scenarios for mixed genders. Our models can also detect stress from ECG specifically for females and males with an average accuracy of up to 79.81% for the females and 73.77% for the males in the low&high combined stress condition, and with the highest accuracy of 81.19% and 75.00% for the females and males in the same condition (see Table 3). Additionally, the average accuracy of the ECG-based stress detection model for the males in the low&high combined stress condition (73.77%) is comparable to that of the mixed genders (74.75%), whereas the average accuracy of the model for the females (79.81%) is higher than that of the mixed genders both in this study (74.75%) and other studies [70, 71]. Based on ECG and EEG (see Table 8), the average classification accuracies of our models for stress detection in the mixed genders, females, and males are as high as 87.75% (high stress), 92.70% (high stress), and 90.08% (low stress), respectively, and with the highest accuracy of 88.75%, 95.00%, and 91.67%, respectively, for the corresponding genders and scenarios (see Table 4).

When performing multilevel stress classification from ECG and EEG using individual classifiers, the average classification accuracy was only 61.00%, 61.62%, and 61.57% for the mixed genders, females, and males, respectively. Using the stacking techniques improved the average classification accuracy for the corresponding genders to 64.08%, 62.60%, and 71.57%, respectively (see Table 8). Note that although very high accuracy values were achieved using deep learning classifiers in [81–83], several signal modalities are required, preventing them from being used in real-world applications.

## Discussion

Stress detection results show that the classification accuracy using only ECG signals is quite satisfactory when considering the ease of sensor development, the level of user-friendly interface, and the complexity of data analysis compared to EEG signals for further development for personal use. However, the combined ECG and EEG signals may be more advantageous when stationary stress evaluation is needed per client visit. Observe that the classification accuracy of our models for stress detection from ECG and ECG together with EEG for the females is higher than that for the males in all the scenarios. This emphasizes that gender is a covariate that should be considered when creating a stress detection model from a physiological signal. The calendar method that was used retrospectively to confirm the menstrual phases of the female subjects revealed that 14 females were in the luteal phase while only 7 females were in the follicular phase of their menstrual cycle. Thus, we hypothesize that since the majority of the females were in the luteal phase of the menstrual cycle, they were not under the protection of high estrogen levels; otherwise, the females would have been less stressed than the males. The higher levels of stress observed in the females compared to those in the males were, as a result, beneficial for the stress detection models in the female subjects.

For multilevel classification, we hypothesize that the gap between the males’ stress levels in the low and high stress conditions was larger than that between the females’ stress levels in the low and high stress conditions, thus making it easier for the stacking technique to differentiate between the low and high stress conditions in the males. The performance of the stacking technique, however, depends heavily on the combination of the base models and their parameters. Our future work will further investigate the effects of different menstrual cycle phases on females’ stress levels and develop portable sensors, such as earphones and a miniature device attached to glasses, and software for real-time stress monitoring. The ability to differentiate between normal states (e.g., sitting still or listening to music) and mental states that are similar to the mental arithmetic tasks carried out in our experiment (e.g., working and writing e-mails) will therefore be beneficial to users for self-monitoring, such that episodes of high stress are kept to a minimum. We will collaborate with psychiatrists to use ECG and EEG-based machine learning models to differentiate between anxiety and depression patients and healthy subjects. We will also develop a meta-model that is less dependent on the parameters of the base models while providing better classification performance.

## Conclusion

*ECG- and EEG-based machine learning models for the detection and multilevel classification of stress for unspecified and specified genders have been developed in this study*. For stress detection, multiple binary models based on ECG can differentiate stress from the baseline in mixed genders, females, and males with acceptable accuracy. For multilevel stress classification based on ECG and EEG, we proposed a combination of LR, NB, ADAB, kNN, RF, SVM, and RBF-SVM classifiers for the stacking technique, which outperformed every individual classifier for mixed genders, females, and males. It must be noted that this work is different from our previous work in several aspects. The models proposed here are capable of both detecting and classifying multiple levels of stress in different genders by constructing models using different parameters and using a dataset that has more features and more subjects. More classification algorithms were also considered, including the stacking technique. To bridge the gap between research and real-world applications, we suggest using the ECG-based model that differentiates between the non-stress and low&high combined stress conditions for personal stress detection applications and the ECG&EEG-based models for stress detection and classification for clinical applications. It has been shown in our study that the difference in gender affects the classification accuracy for both stress detection and multilevel stress classification. This emphasizes the importance of genders for a reliable evaluation of stress and paves the way for building more reliable stress detection and multilevel stress classification models with genders taken into account.
